# Predictors of Return to Work After Stroke in Hungary: A Mixed-Methods Economic and Clinical Data Analysis

**DOI:** 10.3390/healthcare13172198

**Published:** 2025-09-02

**Authors:** Arie Arizandi Kurnianto, Sándor Kovács, Nagy Ágnes

**Affiliations:** Center for Health Technology Assessment and Pharmacoeconomic Research, Faculty of Pharmacy, University of Pécs, H-7624 Pécs, Hungary; kovacs.sandor@pte.hu (S.K.); nagy.agnes5@pte.hu (N.Á.)

**Keywords:** return to work, stroke, rehabilitation, economic evaluation, occupational medicine

## Abstract

Background: Return to work (RTW) is a fundamental aspect of recovery after stroke, importantly, for workers of working age. Evidence indicates there is little known about the clinical and systematic predictors of RTW in Hungary. We aimed to determine the independent predictors of RTW for stroke survivors using aggregate hospital data and expert opinion. Methods: A mixed-method study using aggregated national level administrative data from the Pulvita platform (the National Health Insurance Fund of Hungary) and expert interpretation from the physicians who treat stroke survivors. The data analyzed 13,572 inpatient records for stroke hospitalizations from 2015–2024 across Hungarian counties. Results: Stroke severity, cognitive and psychological recovery, and presence of comorbidities were important clinical predictors of RTW. Rehabilitation intensity was greater for people aged 51–65 years, and work-age men appeared to have slightly better access to rehabilitation compared to work-aged women. Patients accessed more medical rehabilitation services than they did occupational or psychosocial services. Access to rehabilitation services may have varied geographically, with patients in counties such as Budapest and Pest having better access due to higher provider availability and cross-county patient movement. In addition, economic extrapolations from the literature on post-stroke care costs may have introduced bias in estimating annual social productivity losses, reported as EUR 19,953 per patient. Conclusions: Clinical and economic factors both impact RTW potential among stroke survivors in Hungary. Although rehabilitation intensity can indicate likelihood of RTW, the lack of a national RTW program acts as a significant barrier to RTW for stroke survivors. This study suggests a need for integrated rehabilitation and RTW systems, with associated future research linking clinical, economic, and labor market status data to develop effective and efficient policy for stroke survivors.

## 1. Introduction

Stroke is a leading cause of disability in Europe, including Central and Eastern European countries, which continue to report high incidence and mortality rates [1,2,3,4,5], with significant long-term impacts on survivors’ quality of life [6]. In addition, improvements in acute and thrombolytic care have represented significant improvements with regard to stroke-related mortality; however, survivors continue to experience residual impairments that restrict quality of life and social engagement. Individuals with mild to moderate strokes are more likely to return to work compared to those with severe strokes [7,8]. The ability to return to work (RTW) following stroke is an important measure of functional recovery for people of working age, as this reflects not only their neurological recovery, but has strong implications regarding social integration, psychosocial factors, and economic impact. Residual disability such as weakness, neurological deficits, and cognitive deficits have consistently been associated with lower RTW rates. Additionally, ischemic strokes demonstrate better RTW rates than hemorrhagic strokes [9,10,11].

In high income countries, significant strides are being made to document the clinical predictors of return to work (RTW) after stroke. The literature on rehabilitation interventions, especially interventions combining medical, psychological, and vocational elements are promising for improving RTW outcomes, by reducing lost workdays and improving workforce re-integration [12]. Work facilitates social participation and helps maintain social networks, which are crucial for mental health and community reintegration [13]. High rates of RTW among stroke survivors can significantly reduce the economic burden on healthcare systems and social services. It also ensures that individuals continue to contribute to the economy through their work [10]. Clinical predictors of vocational reintegration include stroke severity, cognitive impairment, and post-stroke depression, as well as comorbidities [14].

However, the relation between clinical factors and actual employment outcomes can be difficult to document, as in many cases, patients face non-medical RTW barriers (e.g., inflexible workplace, lack of vocational rehabilitation, and poor social insurance policies). Non-medical barriers (e.g., financial, insurance, and employer flexibility) can be key barriers to RTW for stroke survivors. Addressing financial and non-financial barriers from improved insurance policies, the flexibility of the employer, and educational interventions for employers are important for increasing the likelihood of successful RTW for stroke survivors [15,16,17]. This is especially relevant in Central and Eastern Europe, as the mechanisms and structural supports to re-establish disabled workers may be minimal when compared to Western nations [18]. Nevertheless, cognitive deficits are a major predictor of RTW outcomes. Studies show that the initial cognitive severity of a stroke significantly predicts the inability to return to work [19]. Functional independence, particularly in activities of daily living, is a strong determinant of RTW [20].

Hungary represents an interesting context for studying post-stroke RTW outcomes [21]. Hungary has a single-payer health insurance system offering free healthcare for acute cerebrovascular disorders, which is similar to other CEE countries. However, within Hungary, there are significant microregional socioeconomic differences that impact stroke outcomes [22]. The national health system is notable for providing universal access to interaction with the acute care level and basic rehabilitation services; however, access to comprehensive, multidisciplinary rehabilitation services, especially those that relate specifically to RTW, is inconsistent geographically [23,24,25,26]. There is also no standardized RTW approach across stroke care pathways, and coordination between providing services and employers is sporadic. These system-level misses may contribute to challenges in reintegration for stroke survivors, especially those of working age.

Although there are some studies in the Hungarian administrative health database to establish (and monitor) stroke incidence, treatment, and discharge [27,28,29,30,31], few have examined the relationship between acute post-stroke rehabilitation and return to work (RTW). Admittedly, there are almost no studies linking stroke hospitalization data with RTW or post-acute care. Furthermore, national concern on disability and rehabilitation policy do not include economic evaluations based on clinician input regarding RTW, making it difficult to make evidence-based decisions.

Limited access to integrated data on clinical recovery and employment outcomes limits policymakers’ ability to use evidence in designing interventions. Without data on clinical and economic indicators that predict RTW, rehabilitation resources may be wasted, while populations with the potential to return to productivity may not receive adequate services. The absence of integrated data makes the use of population-level health data and clinical expertise essential to address this knowledge gap and move toward sustainable and affordable solutions [32,33,34,35].

To address this gap, our study will take a mixed-methods approach. First, we will perform a quantitative analysis of anonymized aggregated data from the Pulvita platform to identify clinical and systems determinants of stroke rehabilitation and the nature of the relations to RTW. Second, we are including qualitative information from clinical experts who are directly involved in stroke rehabilitation and the evaluation of work capacity to contextualize the findings and mitigate interpretative bias. Third, we will conduct a targeted review of the literature, so we can argue that our results are being interpreted in the context of existing international and Hungarian evidence. This triangulation of data will increase the validity of our findings and will allow us to more deeply understand the determinants of RTW in the Hungarian context.

## 2. Materials and Methods

### 2.1. Study Design

This research applied a mixed-methods design, combining quantitative analysis of national aggregated clinical data with expert-informed interpretation to explore predictors of return to work (RTW) following stroke in Hungary. The study aimed to identify clinical, regional, and service-related factors associated with RTW among working-age stroke survivors, ultimately to inform health policy and rehabilitation strategies. The study emphasized contextual understanding by complementing data analysis with insights from clinical experts regularly involved in stroke care and RTW assessments.

In this study, we focused on aggregate data concerning working-age stroke patients (15–64 years old) hospitalized between 2015 and 2024, rather than enrolling individual patients. To enrich interpretation of these findings, expert opinions were gathered from Hungarian physicians with significant experience in stroke rehabilitation and occupational health. These professionals, including neurologists and rehabilitation doctors, were consulted informally to offer their perspectives on RTW-relevant factors and to support the interpretation of data patterns emerging from the national health system.

### 2.2. Data Collection

The quantitative dataset were collected via the Pulvita platform, a Hungarian national digital health system that centralizes hospital-based administrative and clinical data. Specifically, the data were extracted in May 2025. The dataset includes 13,572 aggregated records of hospitalizations related to stroke, stratified by age, gender, county, and type of hospital department (e.g., neurology, rehabilitation). It provides total case numbers, discharges, and mortality figures. No personally identifiable information or individual-level clinical data were accessed. Given the aggregate nature of the dataset, ethical approval was not required.

A targeted literature review was necessary to complement the Hungarian data, focusing on international economic evaluations of rehabilitation and RTW-related interventions. It was necessary to conduct a literature search for evidence of direct costs and RTW outcomes in Hungary, as there were no direct cost/RTW outcome data available. The literature evidence was used to provide comparative estimates of rehabilitation costs and cost-effectiveness across European countries, contextualize the Hungarian findings related to international RTW outcomes, and estimate the potential economic impact of productivity loss and savings from RTW, where national data were not available.

### 2.3. Data Analysis

For purposes of analysis, various Pulvita variables in the database (https://prod.eadatlap.hu/PULVITAsysR2_S0/SysLogin/index.php, accessed on 2 May 2025), were recoded into categorical classifications based on international classifications and clinical guidelines. For example, the variable “Further Fate of Patient” was recoded using the severity categories of disability according to the International Classification of Functioning, Disability, and Health (ICF) [36,37,38]. All analyses were descriptive, reflecting the dataset’s aggregated structure. Counts of patients, days, and types of rehabilitation were summarized by age group, sex, county, type of compensation, and type of funding. Ratios were obtained (as percentages of the total patients in that stratum), and population adjusted rates (per 10,000 persons) from population denominators at the county-level provided in the dataset.

Moreover, missing data (e.g., unknown/not determined) were reflected as missing data in the results, and no imputation was performed. Since the dataset was aggregated, and not individual at the level of the data, no inferential statistics were used, rather the comparison of information was descriptive in nature and focused on comparison across cohorts and trends. The qualitative insights provided by expert clinicians (neurology, rehabilitation medicine, occupational health) were also included in interpreting the trends and patterns, which were used to contextualize the findings. The clinicians’ input was triangulated with a focused literature review to strengthen validity and mitigate interpretive bias.

Patients discharged to home care were categorized as mild disability, as these patients were likely functionally independent or safely returning to their home environment. This discharge destination would typically be strongly associated with high potential for RTW. Conversely, referrals to another inpatient medical facility or long-term institutional care were categorized as moderate to severe disability because of the increased dependency on medical or rehabilitative resources. Transfers between intra-hospital wards, for example, were classified as moderate disability because patients relied on ongoing care but were not completely dependent on medical resources. We continued to use this recoding structure to align with ICF domains of activity and participation, which ties into expected vocational reintegration based on functional capacity. Likewise, the “Type of Intervention” variable was recoded into three (3) broad categories based on the ICF rehabilitation domains and guidelines for stroke rehabilitation: medical rehabilitation (those related to physical therapy, neurorehabilitation), occupational/vocational rehabilitation (those related to work training), and psychosocial rehabilitation (those related to psychological support and social integration services). These categorically defined groups allowed for standardized comparisons and interpretations of the various clinical pathways documented in the administrative dataset.

### 2.4. Data Source

All quantitative data used in this study were derived from the national Pulvita platform, representing officially collected inpatient administrative data structured and anonymized case-level summaries from 2015 to 2024. The dataset included stroke-related admissions coded by ICD-10 diagnoses and were disaggregated by location, hospital unit, and age category. This resource reflects Hungary’s national stroke treatment and rehabilitation flows and served as the foundation for identifying clinical and systemic variables relevant to RTW.

### 2.5. Data Integration and Interpretation

In order to provide interpretation of the findings, the study included expert knowledge from clinicians who were directly involved in the stroke rehabilitation process and work capacity evaluations. We conducted semi-structured consultations with three experts: one neurologist, one rehabilitation physician, and one occupational health specialist. Selection was based on ≥10 years of clinical experience in stroke rehabilitation. These experts reviewed the categorizations and provided validation for clinical interpretation of the aggregated data. These informal discussions provided information and insights on how clinicians approach the clinical decision-making process with regards to RTW, how functional recovery and psychosocial factors were incorporated, and related problems in the Hungarian system that affected access to rehabilitation and working with employers. These perspectives contributed to understanding relevant, though unmeasured, aspects of the data and suggested possible explanations for regional differences. The discussions were considered as interpretive input and not formal qualitative data; the individuals’ opinions were not recorded or cited.

This integrated approach allowed for greater nuance to understanding the clinical and systemic predictors of RTW after stroke in Hungary and contributed to the development of evidence-based recommendations that were based both on empirical trajectories, as well as field-level expertise.

## 3. Results

### 3.1. Perspective of Return to Work Program Implementation

Based on an integrative literature review with some occupational medicine specialist interviews, we identified a prediction frameworks for return to work (RTW) after stroke in Hungary. The factors were conceptualized to cover domain-based factors associated with clinical, psychological, workplace, and systemic areas. These interviews showed the need for multi-disciplinary and policy connected approaches.

The conceptual framework produced from the expert consultations is briefly displayed in Table 1, which shows the expressed key domains, predictors, and experiential knowledge that contribute to RTW implementation. Experts placed a strong emphasis on stroke severity, cognition, and motor impairment as clinical predictors of successful RTW. Stroke severity has emerged as the greatest factor and is best evaluated using a range of standardized assessments. However, it was noted that the assessments are not uniformly adopted across institutions and evaluations of functional capacity vary as a result.

Apart from clinical severity, comorbidity factors such as diabetes, hypertension, chronic pain, and post-stroke fatigue were frequently discussed by participants as potential barriers that may delay or reduce RTW capacity. Experts underscored the importance of ongoing surveillance post-stroke and interdisciplinary rehabilitation approaches to lessen these effects.

Additionally, psychological factors were noted by all, including comorbid depression and anxiety and various facets of motivation, but these factors tended to be under-appreciated yet extremely predictive of RTW outcomes. Experts’ greatest concern for impairing RTW potential was with depression specifically, while high resilience, high motivation, and self-efficacy were strong enablers. Experts encouraged the use of psychological assessment and support throughout rehabilitative care.

Workplace characteristics were also central considerations. Stroke survivors engaged in manual work were less likely to successfully return to work than those returning to office or flexible work situations. In preparing to redeploy stroke survivors in a workplace, whether it be full-time, part-time, or suggesting adjusted workloads, such as number of hours or graduated return to work, expert participants noted having openness from their employers with the ability to adjust roles and capacities as key determinants to successful return.

**Figure 1:**
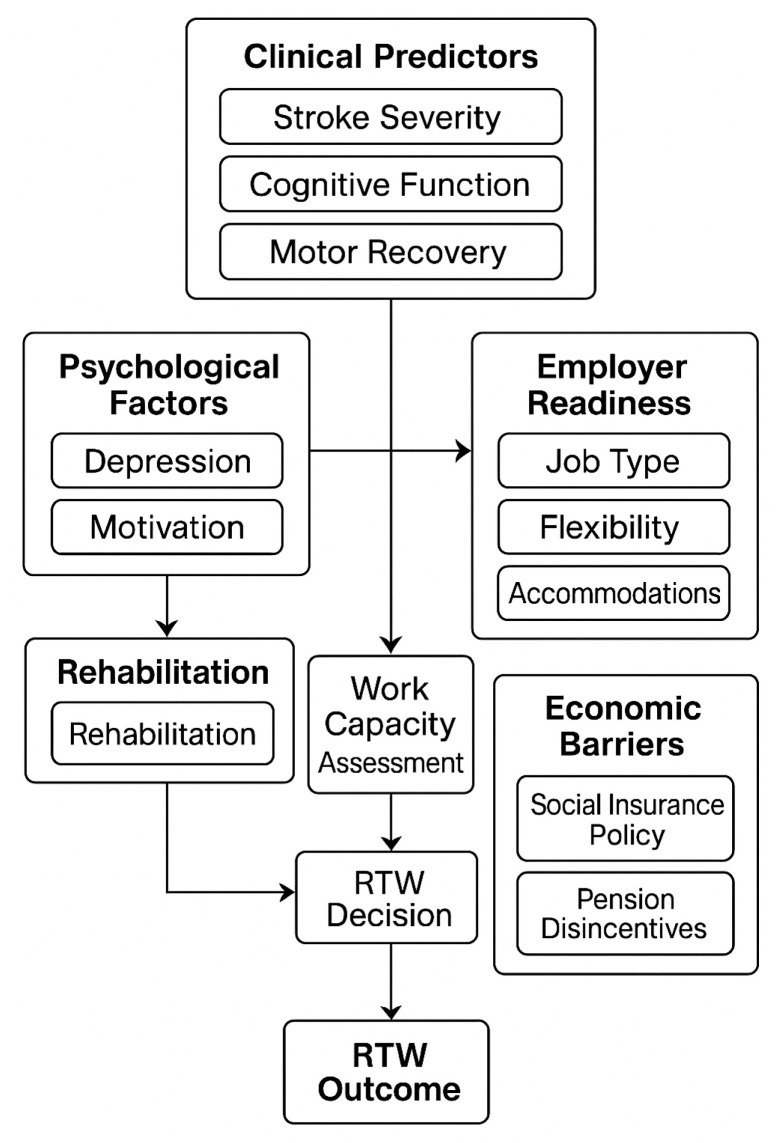
Framework of factors influencing return to work (RTW) after stroke based on a semi-structured interview.

Decisions regarding RTW potential are generally made by interdisciplinary teams of the specialist multidisciplinary team together with the family or caregiver and often based on case-by-case basis with functional capacity examination on file but no standardized national protocol. Expert participants expressed significant reliance on clinical judgement in making therapy recommendations, suggesting a need for formal evidence-based pathways. RTW decision-making generally is construed about member multidiscipline teams by case-by-case evaluations and functional capacity evaluations and generally without standardized national protocols. Experts repeatedly indicated the reliance upon clinical judgement; the anticipation of more structured and evidence-informed pathways is so needed. These interlinked elements are presented in Figure 1, which is a conceptual model of the interaction of clinical, psychological, workplace, and economic factors in determining RTW pathways post-stroke.

Moreover, from an economic perspective, the differences between stroke patients who underwent rehabilitation specifically designed to facilitate return to work (RTW) and stroke patients who had usual care were considerable (see Table 2). Stroke patients who underwent rehabilitation specifically aimed towards RTW had similar and much better outcomes in functional independence (Barthel Index and FIM) and cognitive/psychological outcomes (for example, through the Self-Efficacy Scale), and social reintegration, as compared to stroke patients who underwent usual care, who were more likely to experience persistent impairments and social isolation.

To assist with patient selection and resource allocation, the expert panel supported with the literature also mapped rehabilitation potential in general rehabilitation according to mRS scores (Table 3). Patients with mRS scores of one to three had the most potential for significant recovery and reintegration. Even patients with moderately severe disability (mRS 4) were seen as candidates for improvement, albeit with more limited potential above and beyond the efforts made at rehabilitation. This organizational framework also provides clinicians and policymakers with an ideal pathway for prioritizing and offering RTW rehabilitation services.

### 3.2. Characteristics of Stroke Hospitalization in Hungary

**Figure 2:**
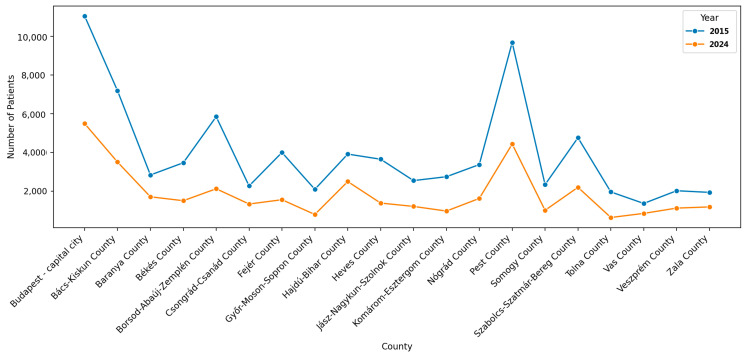
Distribution of patients receiving rehabilitation by county in Hungary, 2015–2024.

The line graph in Figure 2 describes the longitudinal comparison of stroke patients with rehabilitation plans per county throughout Hungary from 2015 to 2024. Each line represents a year—blue for 2015 and orange for 2024—with number of patients per county shown. Budapest proper and Pest County had the highest number of patients in both years, but the drop in numbers from 2015 to 2024 is notable. It is difficult to ascertain the reasons for this decrease, as it may relate to population-based factors. However, the decrease may be related to health system issues where access to alternate care plans became less accessible or available or fewer patients may be referred because policies changed. Other central counties such as Komárom-Esztergom and Bács-Kiskun had significant reductions as well.

Counties with fewer areas had a fewer number (absolute terms) of patients to begin with, and while they did not see a decline as drastic in absolute terms, the proportional decline is nonetheless consistent with this national trend in rehabilitation planning for stroke patients. This decline raises some important questions for policy and health technology assessment—it is unclear if patients are still receiving sufficient rehabilitation, if stroke services are shifting to outpatient/private care, or if there is a real decline in care availability.

**Figure 3:**
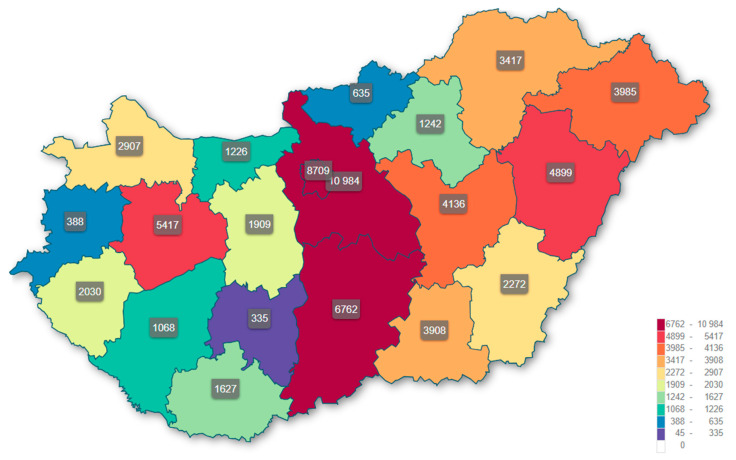
Mapping the number of patients who received a rehabilitation plan after stroke, by county, in Hungary.

Figure 3 shows the number of patients receiving a rehabilitation plan post-stroke, according to county, in Hungary. The central region, especially Pest and Budapest counties, show the greatest number of patients at 10,984 and 8709, respectively, hence indicating a greater access to receiving rehabilitation services or a higher incidence of strokes from a larger population requiring the rehabilitation services. The counties of Bács-Kiskun (6762) and Veszprém (5417) also exhibit a relatively high number of patients receiving an individualized rehabilitation plan. In contrast, the counties of Vas (388), Tolna (335), and Nógrád (635) have a much smaller population of patients, which could have resulted from a lower incidence, underreporting, or less access to structured stroke rehabilitation services. Moreover, from this distribution of the geography of stroke rehabilitation, it is also easy to see potential inequalities in relation to access to services. Meanwhile the more eastern and northeastern counties report relatively high patient counts such as Hajdú-Bihar (4899) and Szabolcs-Szatmár-Bereg (3985); however, they appear to pale in comparison to the counts within central Hungary. A range of theses discrepancies could be linked to urban/rural divides, local health budgets, access of transportation related to services, and socio-demographics.

It is important to recognize that a county’s volume (where patients are receiving rehabilitation) may be affected by distribution of the rehabilitation provider supply across regions, and not simply by incidence of illness. Counties with broader infrastructure for rehabilitation—such as Budapest, Pest, and Bács-Kiskun—would mostly likely take in patients from neighboring counties or from counties with little or no access to rehabilitation services. Subsequently patient domicile and treatment location would possibly be disconnected, and geographic trends in service utilization could also possibly largely reflect clusters of providers and not solely population-based incidence. This may partly account for high rehabilitative counts in the central regions and low numbers in some small or rural counties.

### 3.3. Determinants Prediction of Return to Work After Stroke

Based on triangulation of Pulvita data and clinician input, several key predictors of RTW were identified. Patients discharged home without referral to other inpatient facilities or long-term institutional care had a higher presumed likelihood of resuming employment. Although individual-level economic data were not available, Table 4 shows the extrapolation from the literature, suggesting considerable cost implications.

Acute care costs for the first 12 months after stroke were estimated at between HUF 254,600 and 370,100 depending on age group, while chronic care costs in the second year were estimated at between HUF 36,200 and 50,600. For comparable European countries, inpatient rehabilitation costs averaged EUR 10,530 per case, outpatient rehabilitation averaged USD 17,081, and home program averaged about USD 2306 per patient. The societal costs, predominantly care, were estimated at EUR 19,953 per patient per year, from the perspective of lost productivity, highlighting the long-term economic implications of successful RTW programs.

The Pulvita dataset has carried out a substantial analysis of clinical and demographic factors related to intensive rehabilitation—the potential RTW in Table 5. Rates of rehabilitation were highest amongst ages of 61–65 (34.48%) and 51–60 (30.84%), although mid-life adults (41–50) participated well (26.09%). Male patients were more likely to receive intensive rehabilitation (30.6% vs. 28.8% in females). Patients attending were medically rehabilitated (31.74%); however, vocational (26.41%) and psychological (21.46%) services were under-accessed services and remained important in relation to RTW potential. Most patients receiving rehabilitation were from a Hungarian insurance (Type 1) (29.89%). Although noteworthy, there was a sizable percentage of patients accessing from un-insured packages or alternative reimbursement arrangements (40%), indicating gaps in social access. Engagement rates were highest from non-profit organizations (60.00%) and lower accessed from ecclesiastical and NGOs providers (31.89%), which further reflects the capacity of services.

## 4. Discussion

### 4.1. Key Findings

This study identifies and explores clinical and systemic predictors of return to work among stroke survivors in Hungary based on aggregated administrative and clinical expertise perspectives. We provided evidence that implications of RTW post-stroke may incorporate not only a set of clinical recovery epistemological parameters (like functional states achieved and sustained or stroke severity), but also systemic and organizational contributors mediated by regionally unique systemic resources, including rehabilitation and hospitals. As a general finding, stroke severity measured via hospitalization in neurology and rehabilitation was expected to continue to be the primary prognostic factor in likelihood of return to work. RTW status was not directly available, but the age and gender cohort considerations we provided in Table 5 offer a few simple insights into the composition of sets of factors that may remain relevant in influencing recovery capabilities. Men with stroke generally tended to have greater chances of receiving intensive rehabilitation compared to women. This matches with previous research findings [49,50] in Europe that have observed gendered differences in access to rehabilitation and return to work, where women are likely to experience both extra-occupational social barriers and occupational ones. Based on these data, it is apparent that most working-age stroke patients seeking rehabilitation are male, between the ages of 45–54 years old, and reside in more urbanized counties (which have higher rates of rehabilitation). In Hungary, the working-age population is typically defined as those who are 15–64 years old [51,52].

Importantly, counties like Budapest, Pest, and Bács-Kiskun exhibited significantly higher numbers of patients receiving rehabilitation, which may reflect more comprehensive care availability and better resource allocation. This geographic disparity suggests that health service concentration might enable better vocational reintegration outcomes. The expert perspectives reinforced these findings by identifying cognitive function, mental well-being, and employer involvement as central enablers of successful RTW.

These numbers should be viewed as estimates and should be treated with caution. The disproportionate number of patients in high-volume counties could relate to patient mobility and the unequal distribution of rehabilitation facilities. Those who have just had a stroke may have the option to go to rehabilitation in their own community, but if their county has limited infrastructure, trained professionals, or specialized evidence-based programming, they may seek rehabilitation in a more central community with a better rehabilitation infrastructure. The patient mobility across county boundaries could result in over-reporting of rehabilitation utilization rates in urban centers and under-representing of unmet need existing in peripheral regions.

Additionally, there was variation in the type of rehabilitation provided as well. While medical rehabilitation had quite a large emphasis, there was less emphasis given to vocational and psychosocial rehabilitation, which has a direct relation to RTW, when one compared the overall intensity. If current rehabilitation practice in Hungary leans towards a clinical orientation, which is still meaningful for work-reintegration, it further highlights a structural gap. The analysis by financial type highlighted further heterogeneity, as non-profit organizations had higher rates of intensive rehabilitation by a wide margin. This heterogeneity in provision may also impact equity and access to services that ultimately impact RTW. Future prospective studies should include per capita completion of available facilities and add patient domicile data in order to measure equity and access to post-stroke rehabilitation.

### 4.2. Interpretation in Context of the Literature

Our study supports international evidence that stroke-related functional deficits, as well as cognitive and mood disorders, such as depression, are important predictors of failure to return to work. Prior studies have shown that cognitive and psychological recovery could be stronger indicators of reintegration success than pure motor recovery. While hospital data in Hungary does not provide usable functional scores (e.g., FIM, MoCA or Barthel index), the trends we found in our aggregated data, and verified with expert opinion, followed these patterns. Our findings were also in line with the larger Central–Eastern European context that faced systemic barriers to vocational recovery after stroke, such as fragmented care pathways, limited rehabilitation targeting RTW, and weak employer–healthcare provider integration [5,52,53,54,55].

Hungary has not established a structured national RTW program for stroke survivors. Some countries integrate vocational rehabilitation with the overall continuum of care, commonly with standardized guidelines, employer incentives, and structured follow-up [13,56,57,58,59,60]. The lack of a national structured RTW program in Hungary not only limits reintegration into the workforce but may also contribute to longer-term disability compensation and provide a loss of economic productivity.

### 4.3. Economic Implications

While individual-level cost-effectiveness could not be calculated due to the aggregated nature of the data, the comparative literature from high-income European countries suggests that structured rehabilitation programs are cost-effective when viewed from a productivity and social insurance perspective. For instance, studies from Germany estimate that early post-stroke rehabilitation reduces indirect costs related to lost productivity and disability pensions [45]. In the Hungarian context, the absence of structured RTW pathways may represent a potential missed opportunity for reducing sick-leave duration and supporting labor participation. However, these implications remain tentative given the observational and proxy-based nature of the data, and further research using direct RTW indicators is required. The cost for post-stroke care varies significantly depending on factors such as the severity of the stroke, rehabilitation needs, and dependency levels [61]. Health care expenditure in Hungary was 5.7% of GDP in 2000, which is lower than the average of around 7% in most European countries [62]. This suggests that overall health care funding, including rehabilitation, may be relatively constrained in Hungary. Moreover, in Hungary, cardiac rehabilitation is primarily inpatient, with significant regional disparities in bed availability and utilization [25]. The average length of stay for cardiac rehabilitation increased slightly from 19.2 days in 2014 to 20.2 days in 2017 [25]. Meanwhile, cardiac rehabilitation programs in Europe vary significantly. Western European countries tend to have higher program volumes and more staff compared to other high-income countries [63].

From a policy perspective, the uneven access to rehabilitation services raises issues of efficiency and equity. High-rehabilitation counties are also better aligned with RTW, but it is unclear whether this is because of proactive care models or population size differences. Notably, low per capita rehabilitation cases may simply reflect underutilization, where patients did not access the services available to them. Underutilization may be the result of distance, socioeconomic status, and/or the absence of regional care pathways.

### 4.4. Strengths and Limitations

One of the important strengths of our study is the combination of real-world aggregate hospital data with the unique interpretive perspectives of clinicians who treated patients at a stroke care service or were involved in their return-to-work evaluation. The design of our study as mixed methods allows for triangulation and contextualization of observed trends and moves beyond some of the inherent limitations of administrative datasets.

However, limitations to our study include lack of individual-level follow-up data, no standardized clinical outcome scores (e.g., mRS, NIHSS), and a lack of direct employment outcomes. Product indicators—type of hospital or length of stay—only indirectly indicate readiness for work. Additionally, we were unable to report the data in relation to occupation, type of employment, or compliance with post-discharge rehabilitation. These limitations make it hard to apply our findings directly to cost–utility modelling or policy simulation.

### 4.5. Policy Implications and Future Research

The need for an integrated RTW framework that combines vocational rehabilitation, employer engagement and integrating regionally dispersed stroke rehabilitation services in Hungary has been outlined. Future work should focus on individual-level prospective studies that will link hospitalization with functional recovery and labor market outcomes to support well-designed economic evaluations, such as cost–utility or cost–consequences evaluations.

There is an urgent need to pilot and evaluate structured RTW plans that are stroke specific, ideally in conjunction with national health technology assessment and reimbursement policies. Incorporating occupational rehabilitation and psychosocial rehabilitation as standard components of stroke care could generate significant long-term economic and health benefits.

## 5. Conclusions

This study examined predictors of return to work after stroke in Hungary. In the analysis of aggregated clinical and economic data, key determinants of return to work include age, sex, rehabilitation intensity, and institutional compensation and financing schemas. While direct study of RTW outcomes was not possible because of a scarcity of longitudinal data, we were able to rely on proxy indicators, inferences, and synthesized information from the study; this work offers a valuable understanding of clinical and systemic factors that affect reintegration potential. Future studies should focus on the use of individual level longitudinal data sets to confirm these findings and to inform structured return-to-work programs in Hungary.

## Figures and Tables

**Table 1:** Conceptual framework of stroke patients involved in RTW.

Domain	Key Factors Identified	Insights from Experts
Clinical Predictors	Stroke severity, cognitive function, motor impairment	Stroke severity is the most dominant factor; cognitive and motor recovery are essential for evaluating work readiness.
Comorbidities and Complications	Diabetes, hypertension, chronic pain, post-stroke fatigue	Frequently cited as delaying RTW or limiting capacity, requiring careful follow-up and adjustments.
Psychological Factors	Depression, anxiety, motivation, resilience	Often underestimated but strongly predictive; high motivation aids RTW and depression is a significant barrier.
Clinical Assessments	Barthel Index, return-to-work assessment forms	Used to assess functional status and cognitive capacity; not uniformly applied across institutions.
Workplace Factors	Job type, employer flexibility, accommodations, work environment	Manual labor is less favorable than office work; employer openness is key for successful RTW.
Assessment and Decision Process	Case-by-case evaluation based on functional capacity exams	Clinical judgment and team consensus often guide final RTW decisions.
Rehabilitation and Technologies	Early and continuous rehab, telerehabilitation, vocational therapy	Tech and structured rehab aid reintegration; telerehab helps rural patients but access remains uneven.
Economic Factors	Social insurance rules, employer incentives/disincentives	Some policies may discourage RTW; rigid insurance timelines hinder gradual re-entry.
Barriers in Practice	Lack of employer awareness, rehabilitation capacity, regional disparities	Return-to-work pathways not standardized; rural areas face serious limitations.
Suggestions for Improvement	National RTW framework, incentivizing employers, linking clinical and economic data, better follow-up	Experts support integrated data systems and more flexible, evidence-based policies.

**Table 2:** Comparison of health states and parameters in stroke patients undergoing rehabilitation for return to work vs. usual care.

Parameter	Rehabilitation (RTW)	Usual Care (Non-Rehabilitated)
Functional Independence	High (Barthel Index, FIM)	Low
Cognitive and Psychological	Addressed (Self-Efficacy Scale)	Persistent impairments
Physical Health	Improved (NIHSS)	Prolonged impairments
Social and Occupational Factors	Considered (Employment status, job type)	Neglected, leading to isolation

**Table 3:** Rehabilitation potential by mRS score.

mRS Score	Description	Rehabilitation Potential	Sources
0	No symptoms	No rehabilitation needed	[39,40,41]
1	No significant disability	High potential for full recovery	[39,40,41]
2	Slight disability	High potential for significant improvement	[39,40,41]
3	Moderate disability	Moderate to high potential for improvement	[41]
4	Moderately severe disability	Moderate potential for improvement	[40,42,43]
5	Severe disability	Low to moderate potential for improvement	[39,40,41,43]

**Table 4:** Cost of stroke rehabilitation based on the literature.

Type of Cost	Amount	Source
Acute Care (first 12 months)	HUF 254,000–370,100	[44]
Chronic Care (second 12 months)	HUF 36,200–50,600	[44]
Inpatient Rehabilitation	EUR 10,530 ± 9120	[45]
Outpatient Rehabilitation	USD 17,081	[46]
Home-Based Rehabilitation	USD 2306	[47]
Societal Costs (one year)	EUR 19,953 per patient	[48]

**Table 5:** Clinical determinants of stroke patients.

Predictor Category	Category/Subgroup	Patients with Intense Rehabilitation (%)
Age group	19–30 years old	18.75
	31–40 years old	16.67
	41–50 years old	26.09
	51–60 years old	30.84
	61–65 years old	34.48
	66–70 years old	28.12
Gender	Male	30.61
	Female	28.77
Rehabilitation Type	Medical Rehabilitation	31.74
	Vocational/Occupational Rehabilitation	26.41
	Psychosocial/Psychological Rehabilitation	21.46
Compensation Type	1—free care provided under Hungarian insurance	29.89
	E—provision based on an international agreement based on settlement, provision based on Community rules	10
	D—refugee, asylum seeker	0
	T—care provided to an EU patient by a Hungarian healthcare provider within the framework of cross-border healthcare	0
	Other—reimbursed care for persons who do not have Hungarian insurance or who receive care that cannot be charged to health insurance based on other provisions in force	40
Financial Type	Central budgetary body	31.89
	Non-profit business organization	60.00
	Ecclesiastical legal persons	6.91
	Other business	21.05
	Other NGOs	6.80

## Data Availability

The data that support the findings of this study are available upon reasonable request from the corresponding authors.

## Abbreviations

The following abbreviations are used in this manuscript:

RTW	return to work
ICF	International Classification of Functioning, Disability, and Health
HTA	health technology assessment
NIHSS	National Institutes of Health Stroke Scale
mRS	Modified Rankin Scale
FIM	Functional Independence Measure
MoCA	Montreal Cognitive Assessment
